# Host responses to *Clostridium perfringens* challenge in a chicken model of chronic stress

**DOI:** 10.1186/s13099-020-00362-9

**Published:** 2020-05-06

**Authors:** Sarah J. M. Zaytsoff, Sarah M. Lyons, Alexander M. Garner, Richard R. E. Uwiera, Wesley F. Zandberg, D. Wade Abbott, G. Douglas Inglis

**Affiliations:** 1 Agriculture and Agri-Food Canada, 5403-1st Avenue S, Lethbridge, AB Canada; 2 Department of Agricultural, Food, and Nutritional Science, University of Alberta, 410 Agriculture/Forestry Centre, Edmonton, AB Canada; 3 Department of Biology, University of British Columbia (Okanagan Campus), 1177 Research Road, Kelowna, BC Canada; 4 Department of Biochemistry, University of British Columbia (Okanagan Campus), 1177 Research Road, Kelowna, BC Canada; 5 Department of Chemistry, University of British Columbia (Okanagan Campus), 3247 Research Road, Kelowna, BC Canada

**Keywords:** *Clostridium perfringens*, Physiological stress, Small intestine, Corticosterone, Necrotic enteritis

## Abstract

**Background:**

This study utilized a chicken model of chronic physiological stress mediated by corticosterone (CORT) administration to ascertain how various host metrics are altered upon challenge with *Clostridium perfringens*. Necrotic enteritis (NE) is a disease of the small intestine of chickens incited by *C. perfringens*, which can result in elevated morbidity and mortality. The objective of the current study was to investigate how physiological stress alters host responses and predisposes birds to subclinical NE.

**Results:**

Birds administered CORT exhibited higher densities of *C. perfringens* in their intestine, and this corresponded to altered production of intestinal mucus. Characterization of mucus showed that *C. perfringens* treatment altered the relative abundance of five glycans. Birds inoculated with *C. perfringens* did not exhibit evidence of acute morbidity. However, histopathologic changes were observed in the small intestine of infected birds. Birds administered CORT showed altered gene expression of tight junction proteins (i.e. *CLDN3* and *CLDN5*) and toll-like receptors (i.e. *TLR2* and *TLR15*) in the small intestine. Moreover, birds administered CORT exhibited increased expression of *IL2* and *G*-*CSF* in the spleen, and *IL1β*, *IL2*, *IL18*, *IFNγ*, and *IL6* in the thymus. Body weight gain was impaired only in birds that were administered CORT and challenged with *C. perfringens*.

**Conclusion:**

CORT administration modulated a number of host functions, which corresponded to increased densities of *C. perfringens* in the small intestine and weight gain impairment in chickens. Importantly, results implicate physiological stress as an important predisposing factor to NE, which emphasizes the importance of managing stress to optimize chicken health.

## Background

Poultry are exposed to many stressors throughout production, which can have costly impacts to producers. Necrotic enteritis (NE) incited by *Clostridium perfringens* is an economically-important disease of the small intestine of poultry that results in high bird mortality and costs the global poultry industry US$5–6 billion per year [1]. Research is unravelling the complex nature that physiological stress imparts on disease development, and stressors can both predispose birds to NE and influence the progression of disease [2–4]. However, the mechanisms of predisposition are not well understood.

A number of factors common in poultry production may be involved in predisposition of birds to NE. For example, a co-infection with *Eimeria* spp. predisposes birds to NE by promoting epithelial damage and increasing mucus production, which provides nutrient sources that *C. perfringens* can competitively utilize [5, 6]. Dietary factors, such as the inclusion of fishmeal and wheat/barley in diets, may also be important predisposing factors for disease [5]. Fishmeal has been demonstrated to alter the composition of the microbiota and may provide novel nutrient substrates for *C. perfringens* growth [7]. Wheat and barley are a source of non-starch polysaccharides, which can increase the viscosity of digesta, increase water intake, and result in wet litter [5, 8]. Limited research has examined how stress affects the physiology of birds, and how this impacts *C. perfringens*, and the initiation and progression of NE.

Stress can promote disease via direct and indirect interactions with pathogens. Studies have demonstrated that neurochemicals produced by the host can interact directly with a bacterial pathogen and influence its growth rate and virulence [9]. This has specifically been shown to occur with *Escherichia coli* O157:H7 and *Vibrio parahaemolyticus* where the catecholamine noradrenaline enhanced virulence properties, such as adherence to the intestinal mucosa and increased expression of the type III secretion system [9]. Physiological stress can indirectly promote disease by altering factors within the intestinal environment and modulate immune function. Stress studies in rats have shown that anxiety- and depression-like behaviour increased goblet cell numbers in the intestine [10]. Likewise, in chickens it has been demonstrated that feed withdrawal increased mucin gene expression in the small intestine [11]. Barrier function is another factor that can be altered during physiological stress [12]. For example, early weaning stress and heat stress in pigs has demonstrated reduced transepithelial electrical resistance in the small intestine [13, 14]. Indirect measures of barrier function in chickens have shown that heat stress can alter the expression of tight junction proteins [15]. Additionally, increased bacterial detection in the spleen can occur in birds challenged with *C. perfringens* [6]. Moreover, physiological stress is known to impact immune function in chickens. In this regard, acute stress has been shown to enhance inflammatory responses, whereas chronic stress has resulted in immunosuppression [16]. Repeated stress is particularly important to avoid in production as it results in elevated plasma corticosterone (CORT) levels, promotes immunosuppression through disrupting the Th_1_ − Th_2_/T_reg_ balance, and thereby decreases resistance to disease [16].

Modulations to the composition of the enteric microbiota, physical alterations to the gastrointestinal tract, and changes to the immune status of birds are all potential predisposing states to NE, which can be induced by physiological stress. In the current study we challenged white leghorn chickens with *C. perfringens* and administered CORT in their drinking water as a method to mediate physiological stress. It is noteworthy that various production stressors (i.e. thermal, social, and ammonia) stimulate the production of CORT in chickens [17–19]. However, production stressors are inherently variable. Therefore, we chose to exogenously administer CORT to birds to achieve consistently elevated levels of CORT [20]. This allows for the elucidation of how physiological stress affects the host in a prescribed manner. We contend that this will provide crucial baseline information that will facilitate studies to ascertain the impacts of production stressors on the predisposition of birds to disease. Notably, the exogenously administered CORT model is well established, and it has been used previously to study alterations to host metrics in chickens [20–22]. Using the CORT administration model, a primary goal of the study was to induce a subclinical state of NE to ascertain how extended stress can influence host responses and bird growth. We hypothesize that physiological stress predisposes birds to subclinical NE by promoting the proliferation of *C. perfringens* and modulating the host immune system leading to reduced production performance (e.g. weight gain). Objectives of the study were to determine the impacts of CORT administration on: (i) densities of *C. perfringens* in the intestine; (ii) intestinal mucin production and glycan structure; (iii) tight junction proteins and TLR expression; (iv) disease progression (i.e. histopathologic changes and modulated immune responses); and (v) weight gain in birds. Birds were assigned to one of four following treatments: (1) a negative *C. perfringens* (Cp−) and negative stress (St−) treatment (Cp−St−); (2) a positive *C. perfringens* and negative stress treatment (Cp+St−); (3) a negative *C. perfringens* and a positive stress treatment (Cp−St+); or (4) a positive *C. perfringens* and positive stress treatment (Cp+St+).

## Results

### Corticosterone treatment was associated with increased densities of *C. perfringens* in the small intestine

**Fig. 1:**
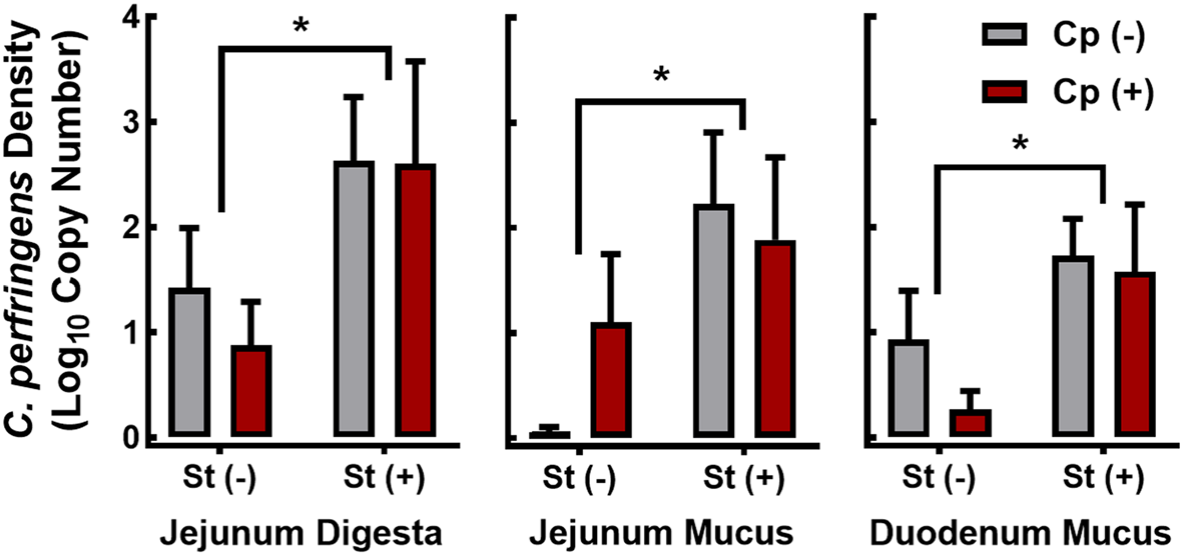
Corticosterone-mediated stress increased densities of NetB and non-NetB *C. perfringens* in the small intestine. Birds were administered 0.2% ethanol drinking water (Cp−St−), challenged with 10^7^ CFU *C. perfringens* (Cp+St−), administered 20 mg/L CORT (Cp−St+), or received both *C. perfringens* and CORT challenge (Cp+St+). Treatments commenced in birds at 14-days-of-age, where *C. perfringens* was administered for 2 days and CORT for 7 days. Densities of *C. perfringens* (log_10_ copies/g mucus or digesta) were determined by quantitative PCR in jejunal digesta, jejunal mucus, and duodenal mucus using primers specific for 16S gene of *C. perfringens*. Vertical lines associated with histogram bars represent standard error of the means (n = 4). Asterisks indicate significant differences (P < 0.050)

Confirmation of the successful colonization of inoculated birds by CP1 *C. perfringens* was confirmed by conventional PCR for the NetB toxin gene, and showed that only inoculated birds (Cp+St− and Cp+St+) were positive for *netB* 24 h post-inoculation (Additional file 1: Fig. S1). Moreover, birds administered CORT (Cp−St+ and Cp+St+) showed increased densities of *C. perfringens* in duodenal mucus (P = 0.035), jejunal mucus (P = 0.033), and jejunal digesta (P = 0.050) in comparison to birds not administered CORT (Cp−St− and Cp+St−; Fig. 1).

### Corticosterone affected mucus production in the small intestine

**Fig. 2:**
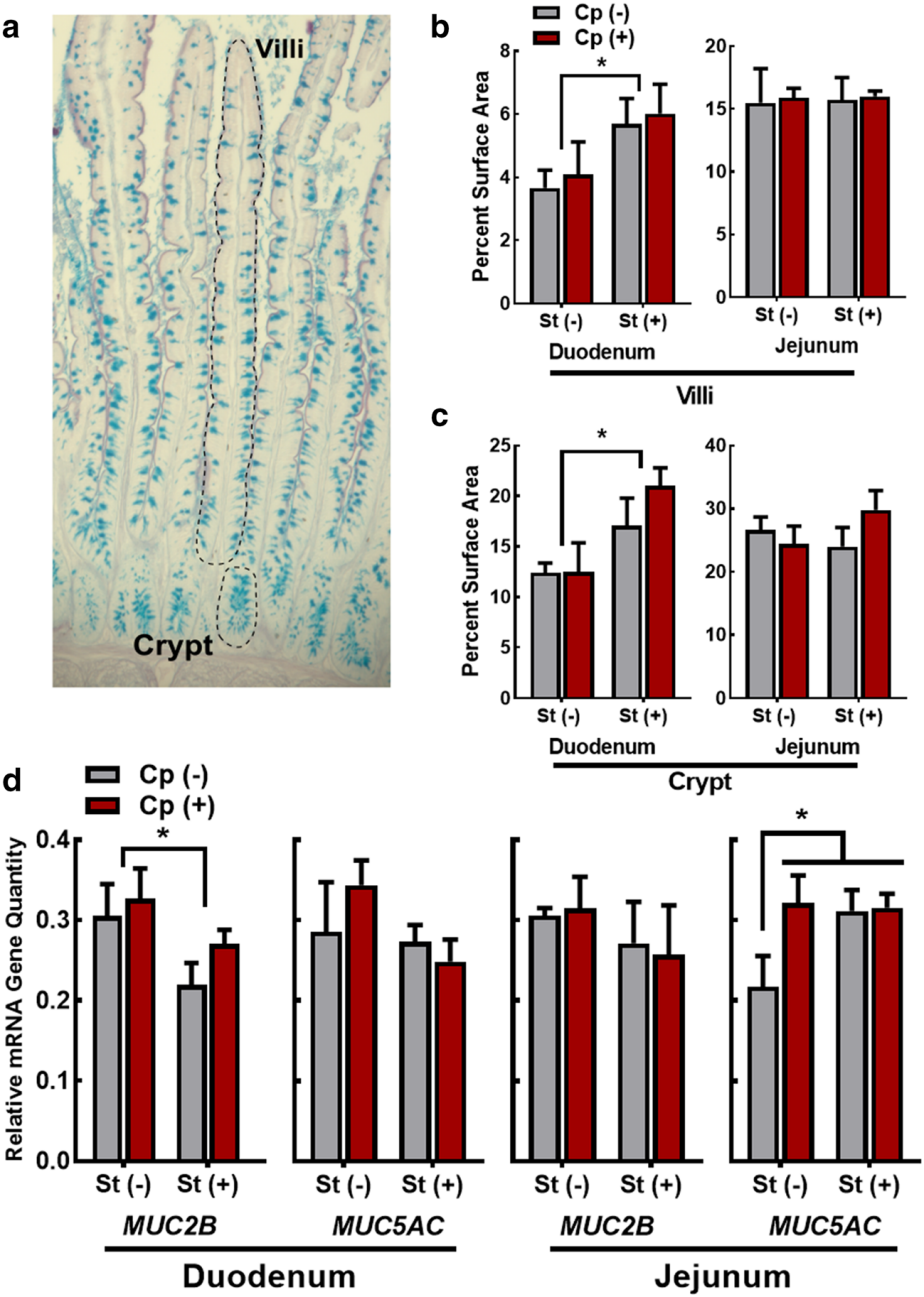
*Clostridium perfringens* infection modulated mucus production. Birds were administered 0.2% ethanol drinking water (Cp−St−), challenged with 10^7^ CFU *C. perfringens* (Cp+St−), administered 20 mg/L CORT (Cp−St+), or received both *C. perfringens* and CORT challenge (Cp+St+). Treatments commenced in birds at 14-days-of-age, where *C. perfringens* was administered for 2 days and CORT for 7 days. **a** Example of a duodenal tissue section stained with alcian blue periodic acid Schiff stain; an individual villus and crypt are indicated with the dotted lines. **b**, **c** ImageJ quantification of alcian blue staining presented as percent surface area of alcian blue staining to total area of villi or crypt region. **b** Villi alcian blue staining in the duodenum and jejunum. **c** Crypt alcian blue staining in the duodenum and jejunum. **d** Relative mRNA gene quantity of *MUC2B* and *MUC5AC* in the duodenum and jejunum. Vertical lines associated with histogram bars represent standard error of the means (n = 4). Asterisks indicate significant differences (P < 0.050)

Duodenal and jejunal tissue sections were prepared and stained with alcian blue. The intensity of alcian blue staining was quantified as a percentage of acidic mucins relative to total size of villi or crypt region (Fig. 2a). Increased mucin staining was observed in the duodenum of birds administered CORT (Cp−St+ and Cp+St+) in both villi (P = 0.039) and crypts (P = 0.012; Fig. 2b). No changes (P = 0.97) were observed in the jejunum. Relative quantities of *MUC2B* and *MUC5AC* mRNA were measured in the duodenum, and decreased (P = 0.045) quantities of *MUC2B* mRNA were observed in birds administered CORT (Cp−St+ and Cp+St+). Quantities of *MUC5AC* mRNA were unaltered (P ≥ 0.67) by both CORT administration and *C. perfringens* inoculation in the duodenum (Fig. 2c). Decreased (P ≤ 0.047) quantities of *MUC5AC* mRNA were observed in the jejunum of Cp−St− treatment birds relative to all other treatments. There was no effect (P ≥ 0.96) of *C. perfringens* or CORT on quantities of MUC2B mRNA in the jejunum.

### Altered mucus glycosylation in *C. perfringens*-infected birds

**Fig. 3:**
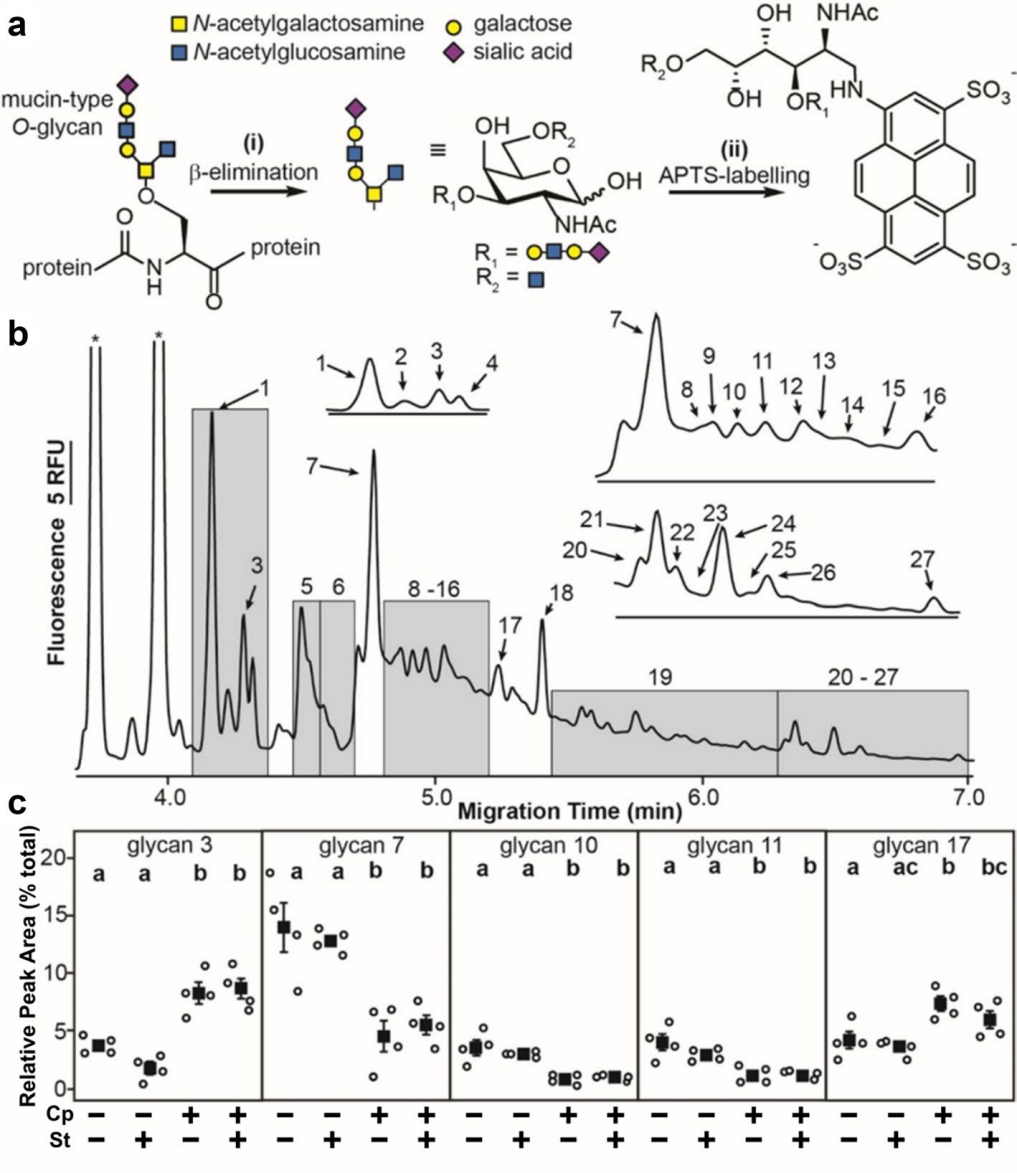
*Clostridium perfringens* induces alterations in mucus *O*-glycan profiles. Birds were administered 0.2% ethanol drinking water (Cp−St−), challenged with 10^7^ CFU *C. perfringens* (Cp+St−), administered 20 mg/L CORT (Cp−St+), or received both *C. perfringens* and CORT challenge (Cp+St+). Treatments commenced in birds at 14-days-of-age, where *C. perfringens* was administered for 2 days and CORT for 7 days. **a** Freeze–dried mucus samples were β-eliminated under non-reducing conditions (i) to yield free, reducing *O*-glycans that could be fluorescently labelled with 8-aminopyrene-1,3,6-trisulfonate, and (ii) resolved by capillary electrophoresis (CE). **b** Resulting CE electropherograms revealed over 50 *O*-glycans per sample, 26 of which were manually integrated in order to calculate their abundances as a percent of the total detected *O*-glycans. Peaks labelled with asterisks are attributable to excess APTS reagent. **c** Strip chart of the *O*-glycans in which relative levels differ (P < 0.050) in one or more treatment group. Solid square markers denote group means, and the vertical lines associated with markers indicate standard error of the means. Markers not labelled with the same letter differ (P ≤ 0.050)

**Fig. 4:**
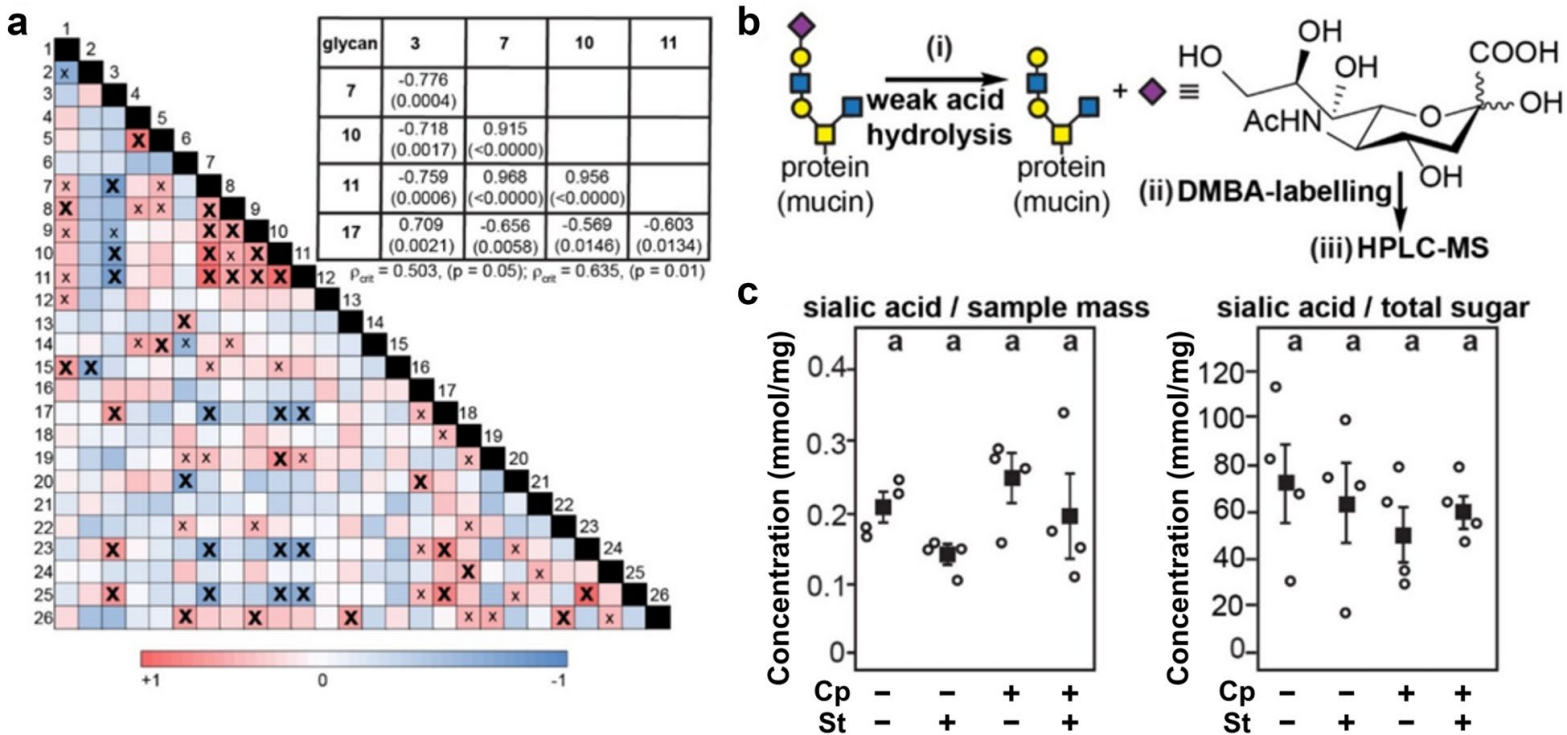
Sialic acid- and sulfate-containing *O*-glycans are inversely correlated, but *Clostridium perfringens* challenge and stress do not significantly affect total sialic acid levels. Birds were administered 0.2% ethanol drinking water (Cp−St−), challenged with 10^7^ CFU *C. perfringens* (Cp+St−), administered 20 mg/L CORT (Cp−St+), or received both *C. perfringens* and CORT challenge (Cp+St+). Treatments commenced in birds at 14-days-of-age, where *C. perfringens* was administered for 2 days and CORT for 7 days. **a** A Spearman’s rank correlation test was performed to determine significant associations between relative levels of all integrated *O*-glycans detected by CE (26) in all mucus samples (n = 16); x = ρ_crit_ < 0.50 (P < 0.050) and X = ρ_crit_ < 0.64 (P = 0.010). **b** Mucus samples were hydrolyzed (i), and labelled with 1,2-diamino-4,5-dimethylbenzene (DMBA; (ii) to permit sialic acid quantitation by HPLC–MS (iii). **c** No significant differences in sialic acid levels were observed among the treatments, although stress tended to lower levels (as a fraction of mucus mass) in both Cp−St+ and Cp+St+ birds. Differing letter codes above each treatment indicate significant differences by the adjusted P-values produced by the HSD test

Total mucin-linked carbohydrates determined by the phenol–sulfuric acid method were 103 ± 25 (Cp−St−), 193 ± 62 (Cp−St+), 78 ± 32 (Cp+St−), and 141 ± 30 (Cp+St+) µmol/mg of mucus. *O*-glycans were chemically liberated from mucins and fluorescently derivatized (Fig. 3a) to enable rapid glycan profiling by Capillary Electrophoresis with Laser Induced Fluorescence (CE-LIF; Fig. 3b; Additional file 1: Fig. S2). Notably, all glycans share a single common fluorophore, rendering the molar detector response independent of glycan structure, and thus permitted substrate-product relationships to be inferred. In all samples, at least 50 glycans of differing electrophoretic mobilities were detected in under 7 min; peak areas for 23 of these glycans were accurately determined Areas for three closely migrating glycans (peaks 5, 6, and 19) could not be reliably assigned, and hence, larger regions were integrated. Many of the *O*-glycans were inferred to be sulfated, given that only peaks 5 and 7 were sensitive to weak acid hydrolysis (indicative of the presence of sialic acids), and given that neutral disaccharides migrate around 5 min, and the addition of sulfate groups decreases migration times by ca. 1 min. Notably, peaks 5 and 7 (and 22 and 21) were also fucosidase-sensitive, while peaks 17, 18, 22, 23 and 24 conspicuously increased in fluorescence intensity after sialic acid hydrolysis (Additional file 1: Fig. S3). Relative abundances of five *O*-glycans were observed to vary (P < 0.050) in birds inoculated with *C. perfringens*; *O*-glycan 3 and 17 both increased (P < 0.001), while 7, 10, and 11 decreased (P < 0.001; Fig. 3c), with over a three-fold decrease in glycan 7 (a fucose and sialic acid-containing glycan) being the most pronounced change. The inverse relationship between glycan 7 (P < 0.001) and 17 (P = 0.007) suggested that these might co-vary. Thus, a Spearman rank correlation test (Fig. 4a) was performed with all 26 glycans for birds in all treatment groups (n = 16). We observed correlations (P < 0.001) among all five glycans that differed (P < 0.050) between *C. perfringens*-inoculated (Cp+St− and Cp+St+) and non-inoculated birds (Cp−St− and Cp−St+). *C. perfringens* can utilize sialic acid as a carbon source in vitro [23]. Coupling this knowledge with the observed inverse correlation between glycan 7 and 17 (P = 0.006), which were also inversely correlated under desialylation conditions (Additional file 1: Fig. S3), total sialic acids (Fig. 4b) were quantitated by HPLC–MS (Fig. 4c). No changes (P > 0.050) in sialic acid levels were apparent, although three of the four birds inoculated with *C. perfringens* exhibited higher than average sialic acid levels per mg mucus, a trend that was reversed by CORT administration.

### Corticosterone affected the duodenal epithelium

**Fig. 5:**
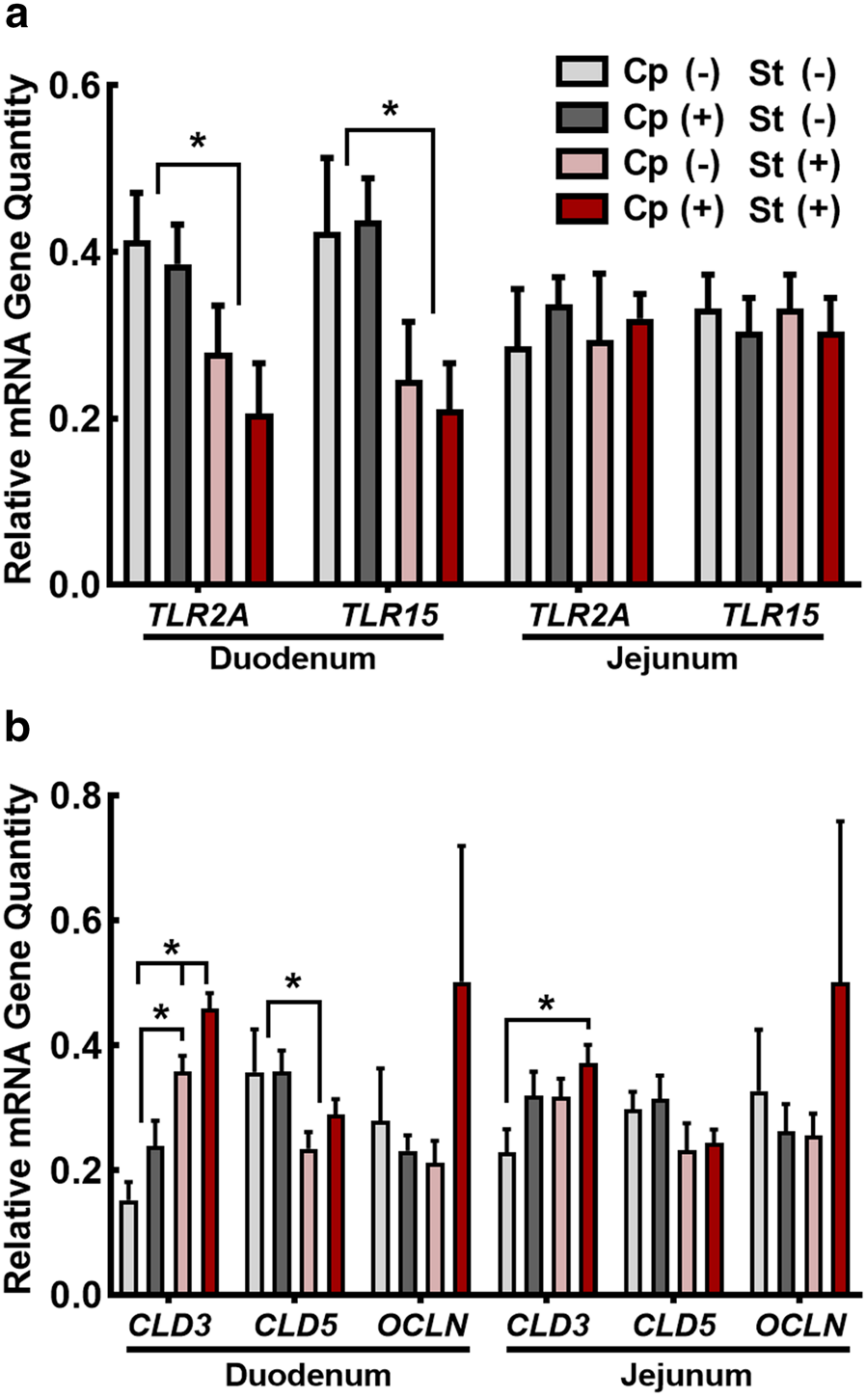
*Clostridium perfringens* challenge and corticosterone treatment modulated relative mRNA gene quantities associated with epithelial function in the small intestine. Birds were administered 0.2% ethanol drinking water (Cp−St−), challenged with 10^7^ CFU *C. perfringens* (Cp+St−), administered 20 mg/L CORT (Cp−St+), or received both *C. perfringens* and CORT challenge (Cp+St+). Treatments commenced in birds at 14-days-of-age, where *C. perfringens* was administered for 2 days and CORT for 7 days. (A-B) Relative mRNA quantities in the duodenum and jejunum. **a***TLR2A* and *TLR15***b***CLDN3*, *CLDN5*, and *OCLN*. Vertical lines associated with histogram bars represent standard error of the means (n = 4). Asterisks indicate significant differences (P < 0.050)

Birds administered CORT (Cp−St+ and Cp+St+) exhibited decreased quantities of *TLR2A* (P < 0.041) and *TLR15* (P < 0.047) mRNA (Fig. 5a). Neither CORT administration or *C. perfringens* inoculation had an effect (P ≥ 0.97) on TLR mRNA in the jejunum. Relative quantities of *CLDN3* mRNA were higher (P ≤ 0.018) in the duodenum of birds administered CORT alone (Cp−St+), and were highest (P < 0.001) in the duodenum of birds inoculated with *C. perfringens* and administered CORT (Cp+St+ ; Fig. 5b). In the jejunum, quantities of *CLDN3* mRNA were only higher (P = 0.011) in inoculated birds administered CORT (Cp+St+) in comparison to Cp−St− treatment birds. Relative quantities of *CLDN5* mRNA in the duodenum were lower (P = 0.042) in birds administered CORT (Cp−St+ and Cp+St+). Neither CORT administration or *C. perfringens* inoculation had an effect (P ≥ 0.11) on quantities of *OCLN* mRNA in the duodenum or jejunum.

### Corticosterone and *C. perfringens* induced histopathologic changes in the small intestine

**Fig. 6:**
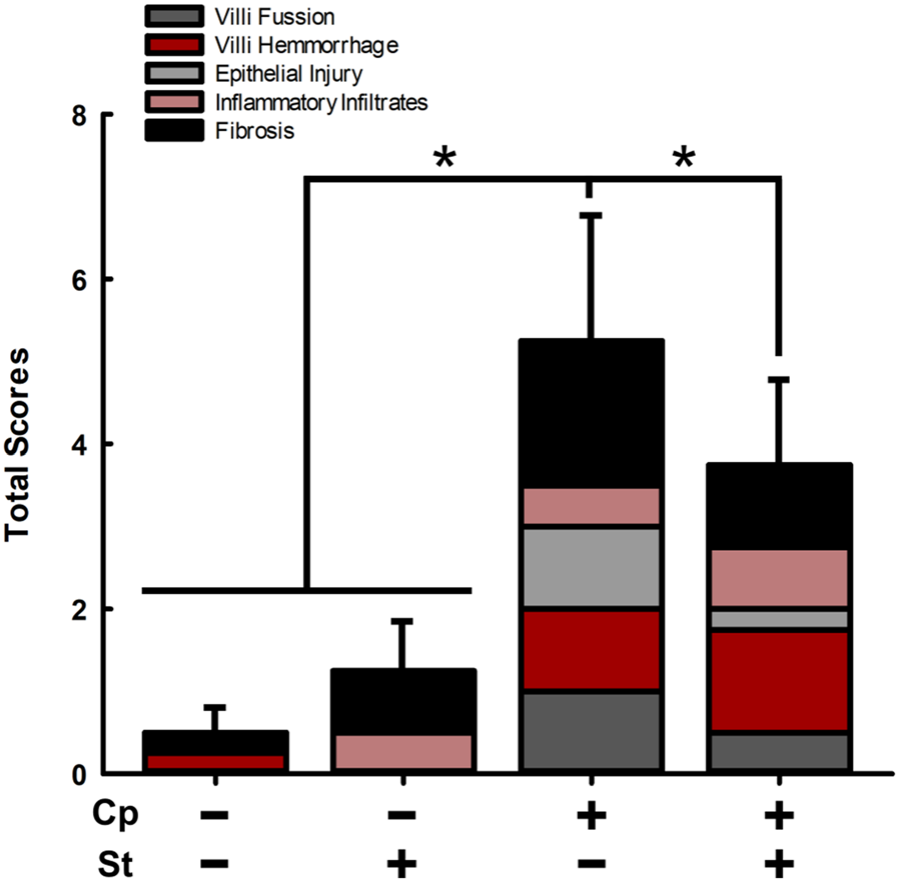
*Clostridium perfringens* incited histopathologic changes in the small intestine. Birds were administered 0.2% ethanol drinking water (Cp−St−), challenged with 10^7^ CFU *C. perfringens* (Cp+St−), administered 20 mg/L CORT (Cp−St+), or received both *C. perfringens* and CORT challenge (Cp+St+). Treatments commenced in birds at 14-days-of-age, where *C. perfringens* was administered for 2 days and CORT for 7 days. Vertical lines associated with histogram bars represent standard error of the means for the total histopathologic score (n = 4). Asterisks indicate significant differences (P < 0.050)

No macroscopic lesions characteristic of NE were observed in the small intestine of any birds. However, histopathologic changes (P < 0.029), albeit it moderate, were observed for birds inoculated with *C. perfringens* (Cp+St− and Cp+St+) in comparison to birds not inoculated with the pathogen (Cp−St− and Cp−St+ ; Fig. 6). Notably, villi hemorrhage contributed to higher (P ≤ 0.050) histopathological scores in Cp+St+ birds in comparison to birds not inoculated with *C. perfringens* (Cp−St− and Cp−St+). Fibrosis was also elevated (P = 0.037) in Cp+St+ birds in comparison to Cp−St− birds.

### Corticosterone and *C. perfringens* modulated immune responses in the spleen and thymus

**Fig. 7:**
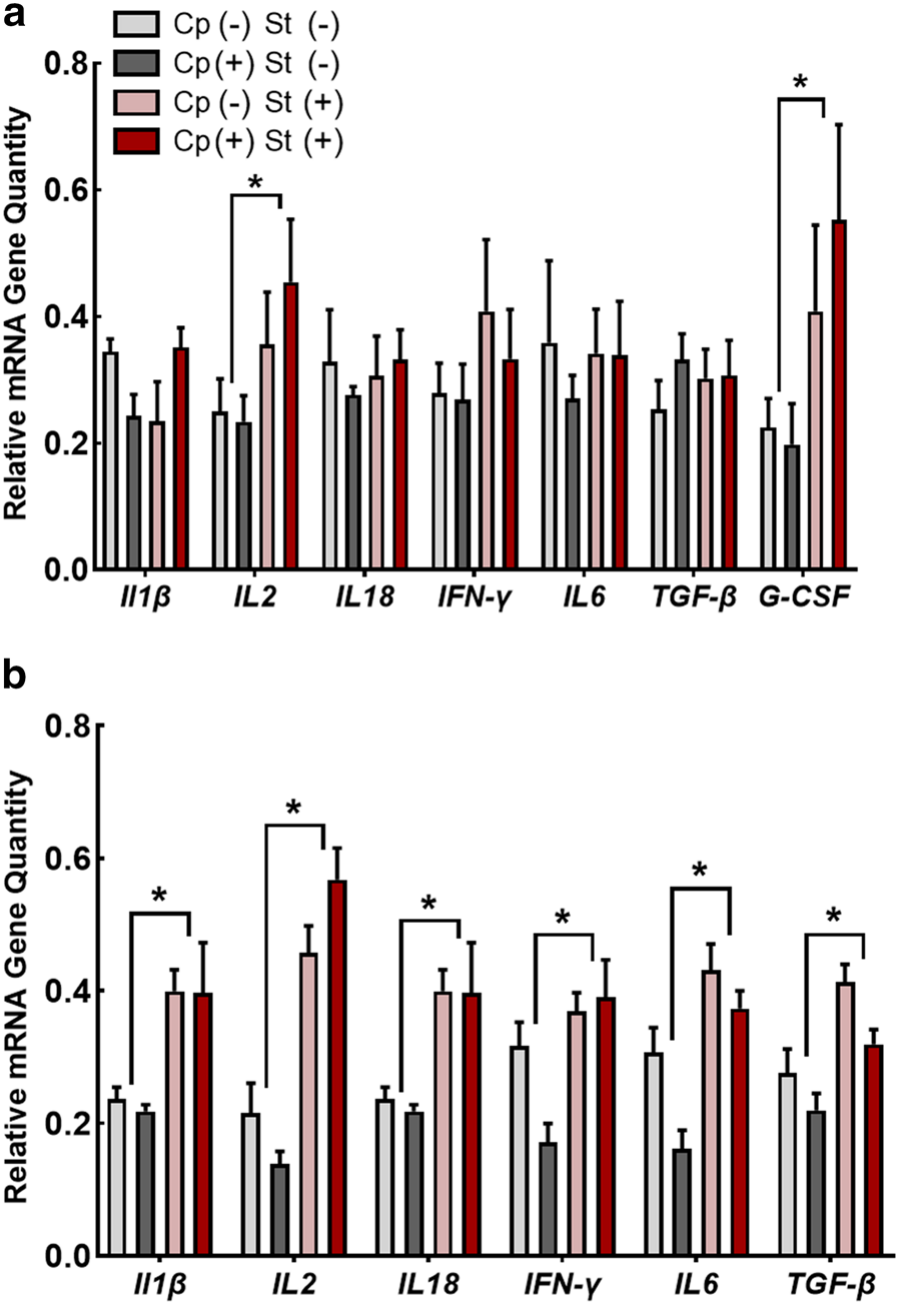
Corticosterone administration modulated relative mRNA gene quantities of immune cytokine genes in the spleen and thymus. Birds were administered 0.2% ethanol drinking water (Cp−St−), challenged with 10^7^ CFU *C. perfringens* (Cp+St−), administered 20 mg/L CORT (Cp−St+), or received both *C. perfringens* and CORT challenge (Cp+St+). Treatments commenced in birds at 14-days-of-age, where *C. perfringens* was administered for 2 days and CORT for 7 days. (A-B) Relative mRNA quantities in the (A) spleen and (B) thymus. Vertical lines associated with histogram bars represent standard error of the means (n = 4). Asterisks indicate significant differences (P < 0.050)

Relative quantities of *IL2* (P = 0.043) and *G*-*CSF* (P = 0.030) mRNA were higher in the spleen of birds administered CORT (Cp−St+ and Cp+St+ ; Fig. 7a). In contrast, CORT administration did not alter (P ≥ 0.19) mRNA levels of *IL1β*, *IL18*, *IFNγ*, *IL6,* or *TGFβ* in the spleen. In the thymus, relative quantities of *IL1β*, *IL2*, *IL18*, *IFNγ*, *IL6*, and *TGFβ* mRNA were higher (P ≤ 0.005) in birds administered CORT (Cp−St+ and Cp+St+ ; Fig. 7b).

### Administration of corticosterone and *C. perfringens* in combination reduced weight gain

**Fig. 8:**
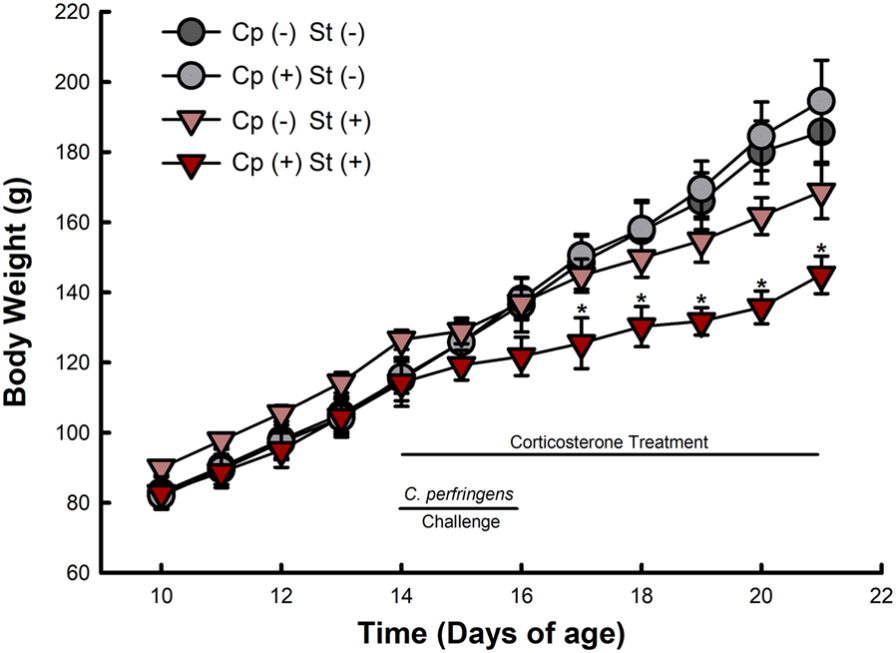
Birds co-challenged with *C. perfringens* and corticosterone exhibited decreased weight gain. Birds were weighed daily from 10 days post-hatch to the study endpoint. Birds were administered 0.2% ethanol drinking water (Cp−St−), challenged with 10^7^ CFU *C. perfringens* (Cp+St−), administered 20 mg/L CORT (Cp−St+), or received both *C. perfringens* and CORT challenge (Cp+St+). Treatments commenced in birds at 14-days-of-age, where *C. perfringens* was administered for 2 days and CORT for 7 days. Vertical lines associated with markers represent standard error of the means (n = 4); markers without vertical lines indicates marker is obscuring the standard error mean or standard error of mean is too small to be represented. Asterisks indicate significant differences (P < 0.050) between the Cp+St+ treatment relative to Cp−St− and Cp+St− treatments

Only birds administered *C. perfringens* and CORT (Cp+St+) exhibited reduced (P ≤ 0.050) weight gain relative to other treatments (Fig. 8). This effect was initially detected (P ≤ 0.015) 3 days after inoculation with *C. perfringens* and commencement of CORT administration, and persisted (P ≤ 0.005) for the remainder of the experimental period.

## Discussion

The mode in which physiological stress predisposes chickens to disease is complex in nature and includes a variety of factors that can be modulated within the host. In the current study, CORT was administered to birds to determine if physiological stress can modulate host responses within the intestine and immune organs in a manner that could predispose them to NE. Additionally, we investigated the impact of *C. perfringens* and physiological stress on body weight gain and subclinical NE.

### *Clostridium perfringens* densities in the small intestine

The risk of NE is elevated when higher densities of *C. perfringens* are present in the intestine of chickens [24]. It has been demonstrated that heat stress, cold stress, and high stocking density in chickens can elevate *C. perfringens* cell densities in ceca [2, 3, 25]. We examined the impact of stress on *C. perfringens* densities in the small intestine as it is the primary site of NE incitation. Notably, we observed that all birds were colonized with non-NetB *C. perfringens* strains that presumably originated from eggs (i.e. the specific-pathogen-free leghorn chickens used in the study are not *C. perfringens* free). However, only birds inoculated with NetB-containing *C. perfringens* were positive for *netB* detection in the feces. We observed that CORT administration was associated with an in increase in NetB and non-NetB *C. perfringens* densities within duodenal mucus, jejunal mucus, and jejunal digesta. Higher intestinal densities of *C. perfringens* result in elevated fecal shedding of *C. perfringens* into the environment which in turn exposes birds to re-inoculation by the bacterium, and horizontal transmission to other birds [25]. Moreover, higher densities of *C. perfringens* may facilitate quorum sensing and increased release of toxins in the intestine [26]. For example, the VirSR two-component system has been shown to transcriptionally regulate the NetB toxin, which is considered to be an important pathogenicity factor in *C. perfringens*. Thus, higher numbers of *C. perfringens* in the intestine would be expected to elevate the probability of NE development.

### Alterations to intestinal mucus

Physiological stress can alter the intestinal environment in a manner that provides novel substrates to microorganisms. In this regard, it has been hypothesized that *C. perfringens* can utilize intestinal mucins as a mechanism to gain access to host epithelium [6]. Recent research by our team has demonstrated that *C. perfringens* is capable of growing on the mucin monosaccharides d-galactose, d-mannose, and sialic acid (*i*.*e*. *N*-acetyl-d-neuraminic acid, and *N*-acetyl-d-glucosamine) when provided as the sole carbon source [23]. Furthermore, selection for *C. perfringens* under enhanced mucus production was observed in piglets [27]. In the current study, we visually measured intestinal mucins by staining them with alcian blue, and quantified the intensity of mucin staining within the small intestine. We observed increased mucin staining in the duodenum in both villi and crypt regions of birds administered CORT. A relationship between *C. perfringens* densities and increased goblet cell numbers has previously been observed in ileal crypts of broiler chickens [6]. We observed that the density of *C. perfringens* was highest in birds administered CORT, which corresponded with the increased mucus production detected in the duodenum of birds. Previous reports have demonstrated increased expression of *MUC5AC* in the jejunum of *Eimeria* spp. challenged birds, and decreased expression of *MUC2B* in birds co-challenged with *C. perfringens* and *Eimeria* spp. [28]. We observed an increase in *MUC5AC* in the jejunum of birds administered *C. perfringens* alone, CORT alone, and *C. perfringens* and CORT in combination. A decrease in the expression of *MUC2B* was only observed in the duodenum of birds administered CORT. Our visual staining of mucins did not correspond with the gene expression results. In this regard, we observed increased intensity of mucin staining within duodenal crypts and villi, but observed no changes in mucin quantities in the jejunum among treatments. One possibility is that measuring mRNA gene expression may not be an accurate way to assess the levels of mucins present in the intestine. Furthermore, we sampled birds 7 days post-infection, which may have been too late to detect many changes in mucin gene expression. It is noteworthy that a previous study also reported no change in *MUC2B* expression after a 7 day *C. perfringens* challenge [29].

Intestinal mucins comprise up to 80% *O*-glycans by mass. Thus, changes in the biosynthesis and/or metabolism of mucin *O*-glycans (i.e. by *C. perfringens* or other intestinal microorganisms) may have resulted in increased levels of acidic- (Neu5Ac- or sulfate-containing) and alcian blue-stained glycans even as mRNA levels for MUCs decrease. We observed a trend for higher glycosylation of mucus in birds administered CORT. This observation corresponded with increased intensity of alcian blue staining in the duodenum. We further assessed alterations to O-glycan profiles by CE-LIF. We observed a relative decrease in glycans containing sialic acid (glycan 7) and/or fucose (glycan 7 and 10) with a concurrent increase in sulfated glycans (glycans 3 and 17) in birds inoculated with *C. perfringens*. It is generally accepted that the sulfation of intestinal mucins confers resistance to microbial degradation [30, 31]. These results indicate that *C. perfringens* could modify the composition of mucins, although it is unknown whether this occurred due to *C. perfringens* utilization of O-glycans, or because host production of mucins was modified due to infection by *C. perfringens*. Future experiments in the presence of sialidase inhibitors will aid in resolving how *C. perfringens* can alter the O-glycan composition of intestinal mucins [32]. In addition, techniques permitting absolute glycan quantitation (rather than assessing relative levels as was done here and elsewhere [33]) will be needed to fully resolve transcriptional and post-transcriptional alterations in mucin biosynthesis.

### Modifications to the small intestinal epithelium

Epithelial properties were examined by measuring mRNA expression of tight junction proteins and TLRs. Claudin 3 and 5 function to maintain barrier integrity by regulating paracellular permeability to ions, solutes, and proteins [34]. We observed that both *C. perfringens* and CORT administration incited elevated expression of *CLDN3* in the duodenum. Therapeutic use of glucocorticoids in humans has been shown to enhance intestinal epithelial tight junction integrity, and this corresponds with increased claudin expression [35]. Conversely, increased expression of *CLDN3* in alveolar epithelial cells has been associated with a decrease in barrier function [36]. Toxigenic intestinal pathogens are capable of disrupting barrier function by altering tight junction proteins [37, 38], and given the toxigenic nature of *C. perfringens*, it is possible this influenced the alterations to claudin expression that was observed in the current study. Heat stress in chickens has been shown to increase *CLDN5* expression in the jejunum and ileum [15]. Likewise, mycotoxin treatment resulted in an increase in *CLDN5*, which may have been the result of subclinical inflammation [39]. In contrast to claudins, we showed that occludin was unaffected by *C. perfringens* and/or CORT administration as has been previously reported in birds exposed to heat stress [15]. Occludin is a tight junction protein that functions in maintaining barrier integrity, although is not always essential for tight junction formation [40].

Toll-like receptor 2 signalling has been implicated in the preservation of intestinal barrier integrity under a state of inflammation [41]. For example, mice with colitis induced by dextran sulfate sodium exhibited ameliorated clinical symptoms and increased intestinal integrity when treated with a *TLR2* ligand [42]. One function of *TLR2* is to activate nuclear factor-κB resulting in the release of inflammatory cytokine and chemokine mediators [41]. Additionally, *TLR2* mediates an anti-inflammatory effect by inducing the release of *IL10* which in turn inhibits macrophage and dendritic cell effector function [41]. In the current study, we measured the expression of *TLR2* and *TLR15* in the small intestine of chickens. *TLR15* is a unique TLR to avian species, which exhibits homology with *TLR2* [43]. Although a specific ligand to TLR15 has not been identified, it has been demonstrated that both Gram positive and Gram negative bacteria can stimulate expression of *TLR15* mRNA [43]. We observed a similar expression pattern for *TLR2* and *TLR15*, which is consistent with a previous report [44]. This suggests that both of these TLRs are regulated or stimulated in the same manner [44]. The administration of CORT to chickens in the current study resulted in decreased expression of both *TLR2* and *TLR15* in the duodenum. Strenuous exercise at high heat has been shown to decrease the expression to *TLR2* in human peripheral monocytes [45]. Likewise, our results are in agreement with a study that showed that heat stress in chickens challenged with *Salmonella enterica* Enteritidis exhibited decreased *TLR2* expression in the spleen and cecal tonsils [46]. Decreased levels of *TLR2* have been associated with impairment of intestinal integrity [46]. Moreover, it has been shown that *TLR2* signalling can preserve the integrity of the tight junction protein, zona occluden 1 under inflammatory stress-induced damage [47]. Given the importance of tight junction proteins and TLRs in the maintenance of barrier function, our results indicate that physiological stress induced by CORT administration can modify tight junction protein and TLR expression in the small intestine.

### *Clostridium perfringens* challenge induces subclinical necrotic enteritis

We observed that neither *C. perfringens* alone or in combination with CORT incited macroscopic lesions indicative of NE in the small intestine 7 days post challenge with the bacterium. Lesion scores in broiler chickens are highest 1 day after the administration of *C. perfringens*, and NE lesions become inconspicuous 7 days post challenge [48]. However, we intentionally used a layer breed of chicken that is less susceptible to NE [49] as the goal of our study was to examine chronic stress on sublethal NE. In addition to examinations for gross pathologic changes, we examined histopathologic changes in the small intestine. We observed conspicuous, albeit moderate microscopic tissue injury in birds treated with *C. perfringens* alone and in combination with CORT. Importantly, *C. perfringens* inoculated birds presented substantially higher total histopathological scores than non-inoculated birds indicative of subclinical NE. These results are in agreement with a study that examined the impact of heat stress on NE. In this regard, Calefi et al. [50] observed that birds challenged with *C. perfringens* exhibited higher histopathological scores, while the application of heat stress decreased histopathological scoring in birds challenged with the pathogen. It is noteworthy that we also observed a small, yet significant decrease in histological scoring of birds subjected to both *C. perfringens* and CORT. This may be due to the immunomodulatory functions of CORT, which can interfere with the production of inflammatory cytokines and prevent the infiltration of heterophils to the mucosa [50].

To further assess subclinical disease incited by *C. perfringens*, we examined the expression of several immune cytokines in the thymus and spleen. We examined responses in a primary and secondary lymphoid organ as immune responses in the intestine have been previously investigated in *C. perfringens* challenge and stress studies [6, 25, 51, 52]. The spleen is a secondary lymphoid organ that plays a central role in the establishment of the immune system in young birds [53]. The chicken spleen hosts a multitude of functions, which include immune surveillance, lymphocyte differentiation, antibody synthesis, formation of complement, differentiation of blood monocytes into macrophages, and is a primary site for immune complex formation [53, 54]. As chickens lack defined lymph nodes, the spleen is an important organ for disease resistance [54]. We observed that the administration of CORT to birds altered the expression of *IL2* and *G*-*CSF* in the spleen. Interleukin 2 functions to activate and proliferate T cells and it is considered to be mediator of a Th1 response [55]. Although we observed elevated *IL2*, the expression of other inflammatory cytokines (i.e. *IL18*, *IFNγ*, *IL6*) were not altered by CORT administration or *C. perfringens* challenge. We observed elevated *G*-*CSF*, also known as colony stimulating factor 3, which may have been regulating the maturation and function of heterophils as has been suggested previously [56]. The elevated *G*-*CSF* that we observed could correspond with reports of corticosterone impacts on birds inducing elevation of heterophil numbers [16]. It is noteworthy that Hong et al. [57] demonstrated elevated *IL1β* and *IL6* in the spleen of broilers as early as 2 days post-infection with *C. perfringens*. Although we cannot exclude the possibility that acute responses occurred in white leghorns, the significant differential expression of immune genes that we observed 7 days after inoculation with *C. perfringens* is consistent with a prolonged sublethal infection.

In contrast to responses in the spleen, we observed substantial modulation of immune genes in the thymus of birds administered CORT. The differing responses observed in these two organs suggest that glucocorticoids impact these organs differently [58]. The spleen consists primarily of mature immune cells that may be more resistant to the actions of glucocorticoids [58]; whereas, the thymus contains immature thymocytes that may be more sensitive to glucocorticoids [58]. Both the bursa of Fabricius and thymus can be highly affected by glucocorticoids through receptor mediated binding that leads to apoptosis [54]. We have previously demonstrated that CORT can induce lymphoid cell depletion, atrophy, and elevate *IL6* and *TGFβ* responses in the bursa [59]. Furthermore, we observed elevated expression of the inflammatory cytokines, *IL2*, *IL18*, *IL1β*, *IFNγ*, and *IL6* in the thymus after 7 days of CORT administration. This may primarily be due to the local effect of thymocytes undergoing glucocorticoid-mediated cell death with macrophages actively removing debris [58].

### Modulation to body weight gain

Clinical NE is recognized as a problem due to high mortalities and losses to farmers. Subclinical disease can also result in tremendous losses as the impacts of disease can go undetected but adversely affect bird performance (e.g. reduced weight gain). Significantly, an impairment in weight gain can translate into significant production losses, as more resources are required to reach slaughter weight. Although white leghorn chickens inoculated with NetB *C. perfringens* were colonized by the bacterium, which incited modest histopathologic changes to the small intestine, we observed no impairment in weight gain. This is in agreement with other NE studies that showed *C. perfringens* colonization did not affect the weight of broiler chickens [51]. However, we observed significant impairment to weight gain in birds infected with *C. perfringens* and administered CORT. Although the precise mechanisms responsible for this observation remain enigmatic, we have previously shown that CORT administration conspicuously affects bird metabolism [59]. Furthermore, mounting an immune response is metabolically costly and it is plausible that the metabolic cost to birds resulting from physiological stress and infection by *C. perfringens* resulted in the reduction in bird weight gain [60]. We observed significant weight gain reduction beginning at 3 days post-infection with *C. perfringens* and initiation of CORT administration. An examination of host responses earlier in a time course should be conducted in future studies. Moreover, temporal determinations of the metabolic cost to birds sub-lethally infected with pathogens under conditions of physiological stress is warranted.

## Conclusion

We demonstrated that chronic physiological stress mediated by CORT administration altered several host measures that corresponded with increased susceptibility to NE in chickens. Several alterations to mucus characterization, epithelial properties, and immune measures corresponded with the administration of CORT. Importantly, CORT administration resulted in increased densities of *C. perfringens* in the small intestine, and impacted bird weight gain in infected birds. Our findings emphasize the importance of controlling stress in production, not only to enhance bird welfare and performance, but also to influence host-microorganism interactions and decrease the predisposition of birds to disease, including subclinical manifestation of NE and possibly other diseases of chickens.

## Materials and methods

### Ethics statement

The study was carried out in strict accordance with the recommendations specified in the Canadian Council on Animal Care Guidelines. The project was reviewed and approved by the Lethbridge Research and Development Centre (LeRDC) Animal Care Committee (Animal Use Protocol Review #1707) before commencement of the research.

### Experimental design

This study was designed as a factorial experiment with two levels of *C. perfringens* challenge (±) and two levels of stress (±) arranged as a completely randomized design with four replicates (n = 16). The experiment was conducted on two separate occasions (i.e. ‘runs’), with two replicates per run. The four treatments were: (1) a negative *C. perfringens* and negative stress treatment (Cp−St−); (2) a positive *C. perfringens* and negative stress treatment (Cp+St−); (3) a negative *C. perfringens* and a positive stress treatment (Cp−St+); and (4) a positive *C. perfringens* and positive stress treatment (Cp+St+).

### Animals

Specific-pathogen-free white leghorn chickens eggs were obtained from the Canadian Food Inspection Agency (Ottawa, Canada). Eggs were incubated in a Brinsea Octagon 40 Advanced Digital Egg Incubator (Brinsea Products Inc., Titusville, FL) according to the manufacturer’s guidelines from incubating chicken eggs. Eggs were maintained at 37.5 °C and 60% humidity with hourly turning of the eggs for the first 18 days of incubation. Thereafter, eggs were set flat for hatching and humidity was increased to 70%. Chicks (1-day-old) were placed in pairs within individually ventilated cages (1862 cm^2^ floor space; Techniplast, Montreal, QC). These cages were operated in containment mode (i.e. negative air pressure flow) to provide bi-directional HEPA filtered air exchange and protect researchers from pathogens, including *C. perfringens*. Autoclaved wood shavings (United Farmers of Alberta Co-operative Ltd., Lethbridge, AB) were added to each cage, and were replaced each morning. Birds were provided continuous free access to a non-medicated commercial starter diet (Hi-Pro Feeds, Lethbridge, AB; Additional file 2: Table S1) and water by nipple drinker. Birds were maintained at 30 °C for 2 days, 28 °C for 2 days, then maintained at 26 °C for the remainder of the experiment on a 16 h light: 8 h dark cycle. Birds were weighed daily starting at 10 days post-hatch.

### Corticosterone administration

The dose and method of CORT administration was determined in a previous study [59]. CORT (20 mg; Sigma Aldrich Inc.) was dissolved in 2 mL of anhydrous ethanol and added to 1 L of drinking water (0.2%). Water containing CORT was prepared fresh each day, and added to animal cages twice daily. CORT control birds were administered water containing 0.2% ethanol. CORT and/or ethanol was added to water when chicks reached 14-days-of-age and continued until the end of the experiment.

### *Clostridium perfringens* inoculation

A pathogenic strain of *C. perfringens* (CP1) was grown in Heart Infusion Broth in an anaerobic atmosphere using a gas pack (Oxoid™, AnaeroGen™, Thermo Scientific™) for 16 h at 37 °C. At 14-days-of-age, birds were gavaged with 500 µL of *C. perfringens* (5 × 10^7^ CFU total) culture grown for 16 h or Heart Infusion Broth (control) for 2 consecutive days. To enumerate cell densities, the broth culture was diluted in a tenfold dilution series, 200 µL was spread onto Columbia agar containing 5% sheep blood, and colonies were counted at the dilution yielding 30 to 300 CFU. Fecal samples were collected 24 h post-inoculation to confirm infection by the CP1 strain, which is positive for the NetB toxin. Fecal DNA was extracted using QIAamp Fast DNA Stool Mini Kit (Qiagen, Inc., Toronto, ON) and subjected to conventional PCR using primers specific to NetB toxin gene (Additional file 2: Table S2).

### Bird euthanasia and sample collection

Fecal samples were collected 24 h post-inoculation with *C. perfringens* using sterile forceps during daily cage cleaning. At the experimental endpoint, all birds were anesthetized, euthanized, and sampled. Birds were anesthetized with isoflurane (5% isoflurane; 1 L O_2_/min) and blood was collected via intracardiac puncture. Birds were euthanatized by cervical dislocation while under general anaesthesia. The abdomen was opened with a ventral midline incision, and the thymus, spleen, duodenum, jejunum, and the ceca were aseptically removed. The intestine was longitudinally opened using a sterile blade, and digesta in the lumen of the jejunum and ceca was removed using a sterile wooden splint. Mucus in the duodenum was gently scraped from mucosa using a sterile glass microscope slide. Tissue samples for RNA analysis were immediately placed within RNA protect Tissue Reagent (Qiagen Inc.). Tissues for histopathologic examination were placed in 10% neutral buffered formalin (i.e. for hematoxylin and eosin (H&E) staining). Intestinal tissues for staining and visualization of mucins were placed in methacarn (60% methanol; 30% chloroform; 10% glacial acetic acid). With the exception of samples for H&E and mucin examination via microscopy, samples were stored at -80 °C until processed.

### Quantitative PCR of *C. perfringens*

Bacterial genomic DNA from duodenal mucus, jejunal digesta, and cecal digesta was extracted using QIAamp Fast DNA Stool Mini Kit (Qiagen, Inc.). Genomic DNA was extracted from a pure culture of *C. perfringens* CP1 strain using DNeasy Blood and Tissue Kit (Qiagen, Inc.). Cell biomass was lysed using enzymatic lysis buffer (20 mM Tris·Cl, pH 8.0; 2 mM sodium EDTA; 1.2% Triton X-100; 20 mg/mL lysozyme) instead of the lysis solution (buffer ATL) supplied in the kit. A standard curve of known copies of 16S DNA specific to *C. perfringens* was generated with DNA amplified from the CP1 strain (primers in Additional file 2: Table S2). Amplicons were visualized in a 2% agarose gel, and the amplicon was extracted using QIAquick Gel Extraction Kit (Qiagen Inc.). To generate a standard curve of known gene copies, the gel-extracted DNA was quantified fluorometrically using Qubit™ 2 (Life Technologies, Burlington, ON, Canada), and copies of genes were normalized to 10^7^ copies/µL based on concentration, amplicon size, and nucleotide weight. A standard curve was generated by diluting DNA in a tenfold dilution series and amplifying *C. perfringens* 16S DNA using CP1.2 primers (Additional file 2: Table S2). Quantitative PCR (qPCR) was used to measure *C. perfringens* densities in duodenal mucus, jejunal digesta, and cecal digesta relative to the standard curve and normalized by the weight of the sample. Each reaction contained 10.0 µL QuantiTect SYBR green master mix (Qiagen Inc.), 1.0 µL of each primer (10 µM), 2.0 µL bovine serum albumin (1 mg/mL), 4.0 µL DNase-free water, and 2.0 µL template DNA. Reactions conditions were: 95 °C for 15 min; and 40 cycles of 95 °C for 15 s, 55 °C for 30 s, and 72 °C for 30 s; and melt curve analysis from 55 to 95 °C. A Mx3005p thermocycler (Agilent Technologies, Santa Clara, CA) was used to conduct qPCR analysis. Reactions were run in triplicate, and the mean of the three observations was calculated.

### Alcian blue periodic acid Schiff stain and ImageJ quantification

Duodenal and jejunal tissues were fixed in methacarn for a minimum of 48 h. Samples were dehydrated using a Leica tissue processor (Leica TP1020 Benchtop Tissue Processor, Leica Biosystems, Concord, ON), embedded in paraffin blocks using a Shandon Histocentre 3 Embedding Center (Thermo Scientific, Ottawa, ON), and sectioned (≈ 5 µm) using a Finesse 325 Manual Rotary Microtome (Thermo Scientific). Slides were deparaffinised with xylene and rehydrated to water through a series of decreasing ethanol washes. Slides were stained with 1% alcian blue (pH 2.5, 3% acetic acid) for 30 min and subsequently rinsed with water for 8 min. Slides were immersed into 0.5% periodic acid for 10 min followed by 5 min washing with water, stained with Schiff solution for 20 min, and rinsed with warm tap water for 10 min. Slides were then dehydrated with 100% ethanol, cleared with xylene and cover slipped with Permount™ mounting medium (Ficher Chemical SP15500). Each tissue sample was visualized with a Zeiss Axioskop2 Plus microscope (Ziess Canada Ltd., Toronto, ON), and photographed using an Axiocam 506 color camera (Ziess Canada Ltd.) with Zen 2.0 software. The surface area of alcian blue staining was quantified in eight arbitrarily-selected intact villi and crypts for each tissue section. To ensure uniformity in staining and analysis, all slides were stained and photographed at the same time. The individual completing quantification analysis was blinded to treatments. Alcian blue staining was quantified using ImageJ as previously described [6, 61]. Briefly, each picture was imported into ImageJ and made into a RGB stack. The red stack was used for quantification where lower threshold was set to zero and upper threshold set to 140. The region of interest tool was used to select either the villi or crypt region. The measure function was used to obtain percent surface area of alcian blue staining in the region of interest.

### Characterization of mucus

#### *O*-glycans

Mucus was transferred to pre-weighed 1.5 mL screw-capped centrifuge tubes (Sarstedt, Germany), dried by lyophilisation (reweighed), and subjected to ammonia-catalyzed β-elimination as previously described [62] with several modifications. In brief, samples were heated in (NH_4_)_2_CO_3_ (Sigma Aldrich) saturated NH_4_OH (Anachemia, VWR, Montrel, QC) for 40 h at 60 °C. After cooling, samples were evaporated to dryness at ambient temperature using a SpeedVac (Thermo Fisher Scientific™) concentrator; 1 mL of 18 MΩcm^−1^ water was added to the dried samples which were mixed by vortexing and sonication in a water bath sonicator (VWR, Edmonton, AB), and dried a second time to remove ammonium salts. Samples were suspended in 100 μL of 18 MΩcm^−1^ water and insoluble proteins were pelleted by centrifugation (10,000×*g*, 5 min, 21 °C). Without prior acidification or reduction, the released *O*-glycans contained in the supernatant were subsequently desalted using 250 mg Supelco ENVICarb solid phase extraction (SPE) cartridges (Sigma Aldrich) [23]. Desalted *O*-glycans were labelled with 8-aminopyrene-1,3,6-trisulfonate (APTS) and subsequently resolved by capillary electrophoresis (CE; ProteomeLab PA800; Beckman-Coulter); APTS-labelled glycans were detected by laser-induced fluorescence (LIF) [63, 64]. Peaks were manually integrated using 32Karat Software Version 7.0 (Beckman-Coulter); in several instances, peaks migrated too closely together to enable accurate comparisons/integrations between different samples, and in these cases, whole regions of the electropherograms were integrated. All glycan peak areas were expressed relative to the sum of the total integrated areas of peaks not attributable to the APTS labelling reagent. APTS-labelled *O*-glycans were treated with bovine kidney α-fucosidase (Sigma Aldrich) or mild acid hydrolysis (see below) in order to identify fucose- and sialic acid-containing glycans, respectively; in each case, loss of a CE peak (relative to a non-treated control) after treatment was taken as evidence for these monosaccharide constituents.

#### Sialic acid quantitation

Lyophilized mucus samples were accurately weighed, suspended in 2 M acetic acid (EMD Millipore, Darmstadt, Germany), and subjected to mild acid hydrolysis to selectively liberate sialic acids exactly as previously described [65]. The released sialic acids were derivatized using 1,2-diamino-4,5-dimethylbenzene (DMBA; Sigma Aldrich) and quantitated by high-performance liquid chromatography-mass spectrometry (HPLC–MS) using an external calibration curve. The amount of sialic acid present in each sample was either normalized to the dry sample mass or to the total monosaccharide content as determined by the phenol–sulfuric method [66].

### Quantification of mRNA of response genes

Total RNA was extracted from duodenal, jejunal, splenic, and thymic tissues using a RNeasy mini kit (Qiagen Inc.) as described previously [59]. An additional DNase step was included to remove residual genomic DNA. A Bioanalyzer RNA 6000 Nano kit (Agilent, Mississauga, ON, Canada) was used to measure RNA quality and quantity, and 1 μg of RNA was reverse transcribed to cDNA using a QuantiTect reverse transcription kit (Qiagen Inc.). Quantitative PCR was performed using an Mx3005p thermocycler (Agilent Technologies). Each reaction contained 5.0 µL of QuantiTect SYBR green master mix (Qiagen Inc.), 0.5 µL of each primer (10 µM), 3.0 µL RNase-free water, and 1.0 µL cDNA. PCR conditions were: 95 °C for 15 min; 40 cycles of 95 °C for 15 s, 58 °C for 30 s, and 72 °C for 30 s; and melt curve analysis from 55 to 95 °C. Primer sequences specific to gene targets (Additional file 2; Table S2) were generated using NCBI primer BLAST; primers were designed to create an amplicon between 75 and 200 base pairs. Efficiencies for all primers were between 95 and 110% and a single peak was present in melt curve analysis. Reactions were run in triplicate and the average Ct values were used to calculate gene expression relative to two reference genes (*BA* and *TBP)* using qBase + software (Biogazelle, Gent, Belgium) [67].

### Small intestinal histopathology

Jejunal tissue samples were fixed in formalin for a minimum of 24 h. Samples were dehydrated, embedded in paraffin, and sectioned as described above. Slides were de-paraffinized with xylene and stained with hematoxylin and eosin. Jejunal sections were scored by veterinarian pathologist (RREU) blinded to treatments using a modified scoring criteria previously described [68, 69]. Sections were graded 0 to 4 for villus fusion, villous hemorrhage, epithelial cell injury, red blood cells within lumen, proteinaceous material within the lumen, intestinal inflammatory infiltrates, and fibrosis. The total pathological score was determine by calculating the sum of scores from all categories for each bird.

### Statistical analysis

Statistical Analysis Software (SAS Institute Inc. Cary, NC) was used to perform the majority of statistical analysis except as otherwise noted. With the exception of the histopathologic score data, continuous data was assessed to ensure normality. Treatment and interaction among factors were determined using a mixed linear model. Animal body weight data was treated as a repeated measure; the appropriate covariance structure was utilized according to the lowest Akaike’s Information Criterion. In the event of a significant main effect (P ≤ 0.050), the least squares means test was used to evaluate differences among treatments for qPCR, gene expression, mucin stain intensity, and body weight gain. Categorical data (i.e. histopathologic changes) was analyzed using a non-parametric Fisher’s exact test where pairwise comparisons were performed between all treatments. Differences in glycan abundances were analyzed using R (Version 3.6.1). The normality and homogeneity of variances in the average relative (%) abundances of each *O*-glycan was assessed using normal quantile plots and a Levene’s test, respectively. Analysis of variance (ANOVA) was subsequently employed to determine if there was a difference (at a significance level of 0.050) in *O*-glycan levels associated with each bird treatment. A Tukey’s honestly significant difference (HSD) test was performed to determine statistically significant differences among the means (P < 0.050). A Spearman’s rank correlation test was performed (using Microsoft Excel) to discover co-varying *O*-glycans. Data are represented as the mean ± standard error of the mean.

## Supplementary information

**Additional file 1.** Additional figures.

**Additional file 2.** Additional tables.

## Data Availability

The datasets generated during and/or analysed during the current study are available from the corresponding authors on reasonable request.

## Abbreviations

CORT: Corticosterone
NE: Necrotic enteritis
Cp−: Birds not inoculated with *C. perfringens*
Cp+: Birds inoculated with *C. perfringens*
St−: Birds not administered corticosterone
St+: Birds administered corticosterone
CE-LIF: Capillary electrophoresis with laser induced fluorescence
TLR: Toll-like receptor
CLDN: Claudin
OCLN: Occludin
IL: Interleukin
G-CSF: Granulocyte colony stimulating factor
IFNγ: Interferon-γ
TGFβ: Transforming growth factor β
MUC: Mucin
HPLC-MS: High-performance liquid chromatography-mass spectrometry
qPCR: Quantitative polymerase chain reaction
CFU: Colony forming unit
H&E: Hematoxylin and eosin
Neu5Ac: *N*-acetyl-d-neuraminic acid
SPE: Solid phase extraction
APTS: 8-aminopyrene-1,3,6-trisulfonate
