# Supplementary material for: Host responses to Clostridium perfringens challenge in a chicken model of chronic stress
Source: Gut Pathog. 2020 May 6;12:24. doi: 10.1186/s13099-020-00362-9 (PMC7203818; doi:10.1186/s13099-020-00362-9)
Supplement: Supplementary file 1 — Additional file 1. Additional figures. [file 13099_2020_362_MOESM1_ESM.pdf]

Host responses to *Clostridium perfringens* challenge in a chicken model of chronic stress

Sarah J.M. Zaytsoff,[a,b] Sarah M. Lyons,[c] Alexander M. Garner,[d] Richard R.E. Uwiera,[b] Wesley F. Zandberg,[c,e] D. Wade Abbott,[a] G. Douglas Inglis[a]#

[a]Agriculture and Agri-Food Canada, Lethbridge, Alberta, Canada

[b]Department of Agricultural, Food, and Nutritional Science, University of Alberta, Edmonton, Alberta, Canada

[c]Department of Biology, University of British Columbia (Okanagan Campus), Kelowna, British Columbia, Canada

[d]Department of Biochemistry, University of British Columbia (Okanagan Campus), Kelowna, British Columbia, Canada

[e]Department of Chemistry, University of British Columbia (Okanagan Campus), Kelowna, British Columbia, Canada

SUPPLEMENTAL MATERIAL

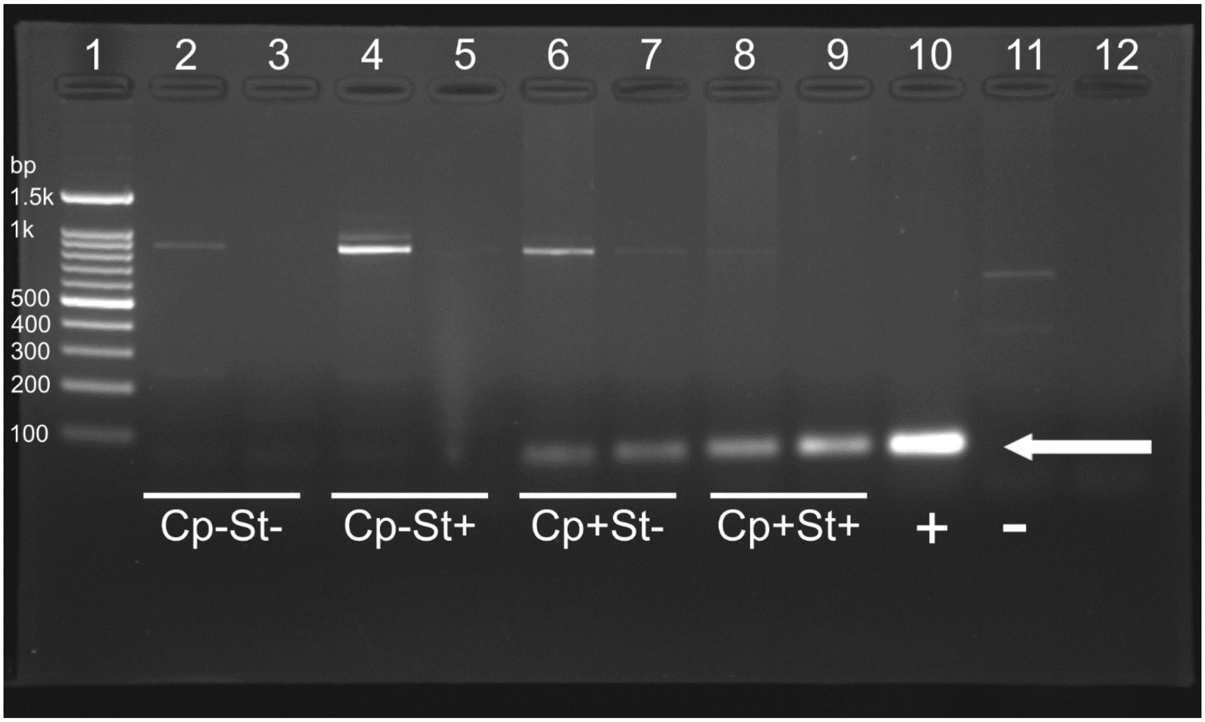

Fig. S1 A 2% agarose gel of NetB gene PCR product (78 bp). Fecal samples were collected 24 h post inoculation with control broth or CP1 *C. perfringens* (NetB toxin positive), DNA was extracted, and subjected to conventional PCR. Lane 1: SMOBIO 100 bp DNA Ladder; Lane 2/3: PCR product of negative control birds (Cp-St-); Lane 4/5: PCR product of birds receiving 20 mg/L corticosterone (Cp-St+); Lane 6/7: PCR product of birds receiving *C. perfringens* inoculation (Cp+St-); Lane 8/9: PCR product of birds receiving both *C. perfringens* inoculation and 20 mg/L corticosterone (Cp+St+); Lane 10: PCR of positive control containing DNA isolated from CP1 *C. perfringens* strain; Lane 11: PCR of negative control containing DNA isolated from confirmed NetB negative *C. perfringens*; and Lane 12: PCR of negative control water blank. Positive bands for NetB gene amplification are seen only in samples obtained from Cp+St- and Cp+St+ treatment groups.

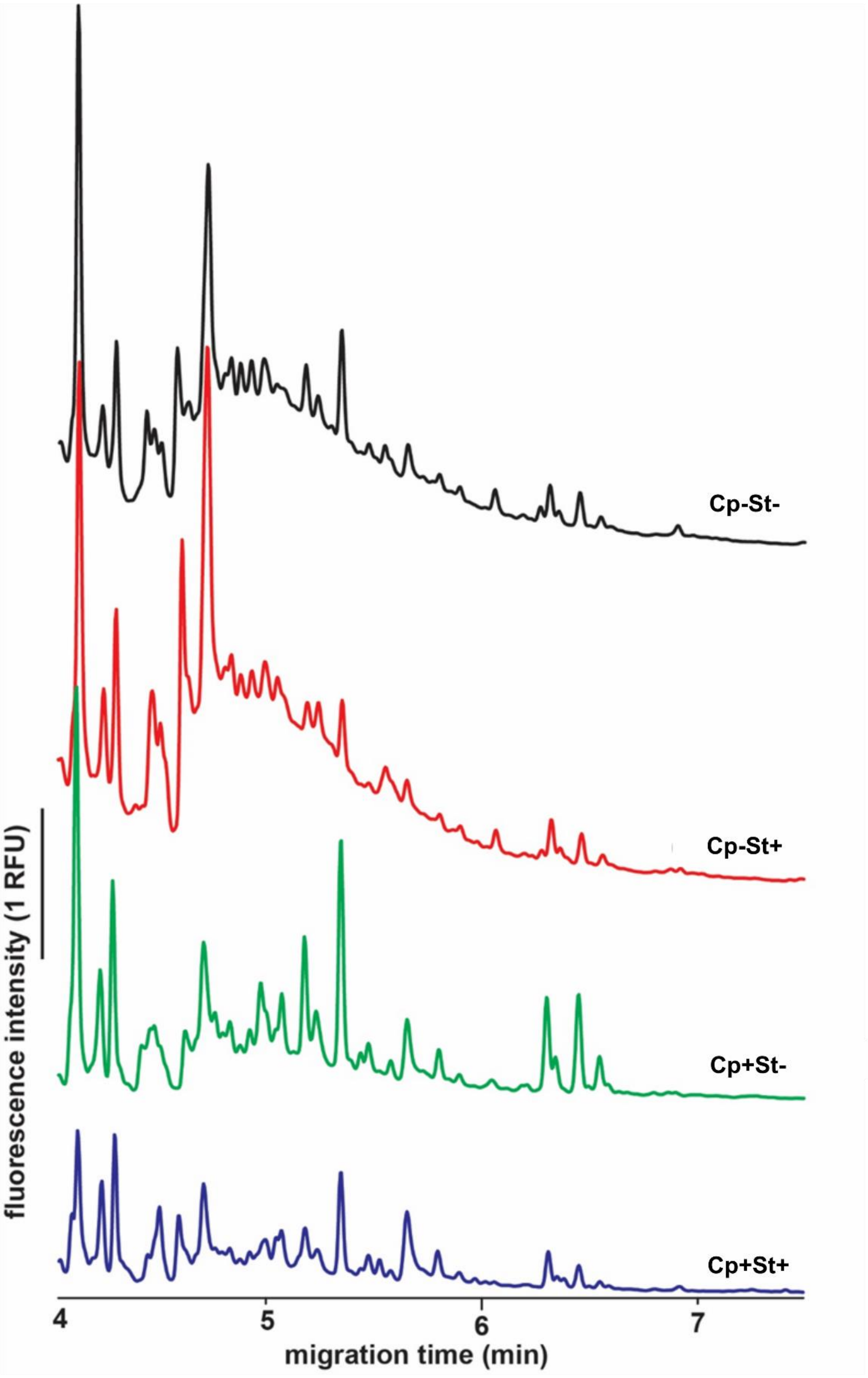

Fig. S2 Representative CE electropherograms of APTS-labelled *O*-glycans derived from intestinal mucus of chickens in the four treatment groups.

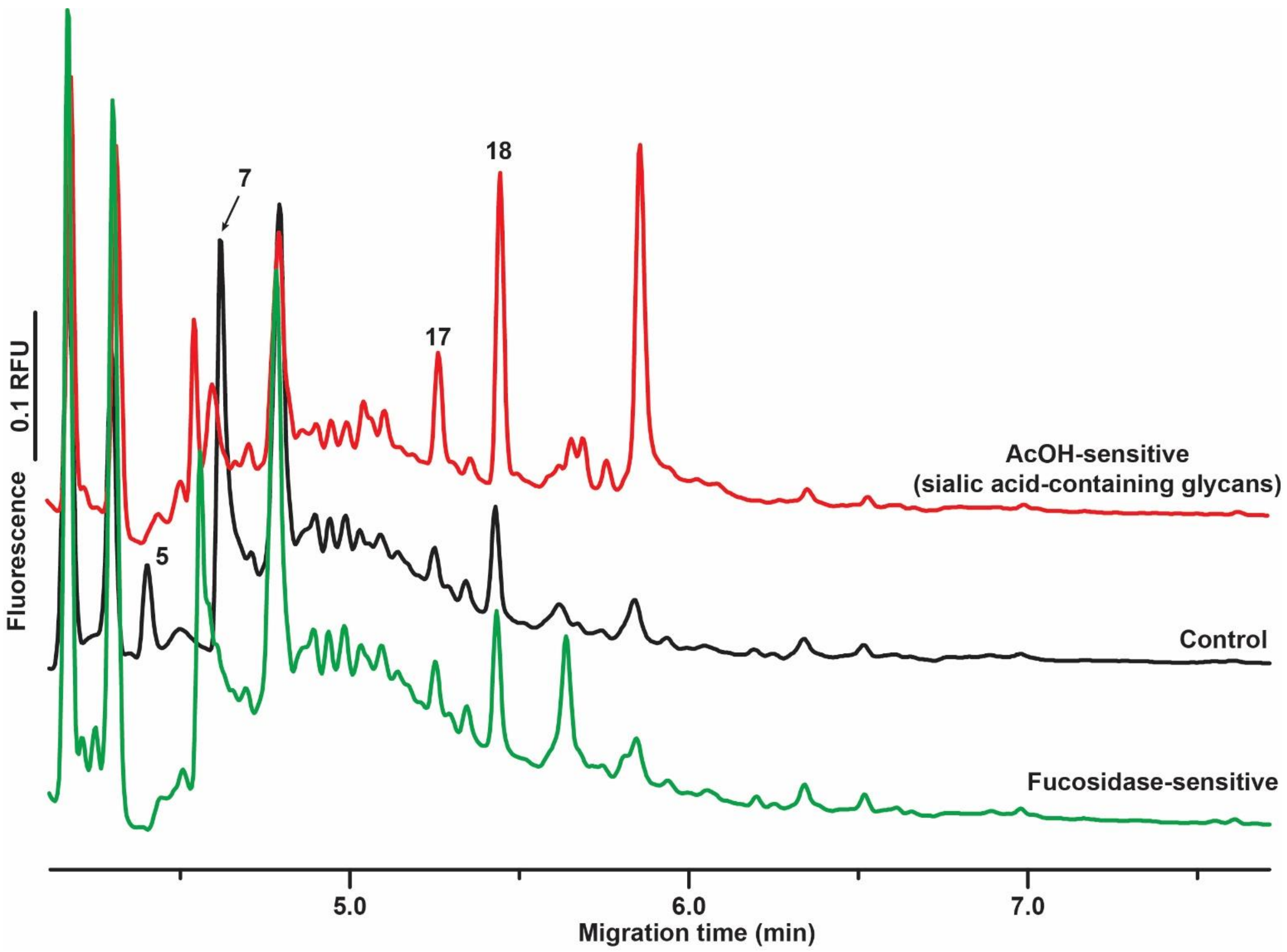

Fig. S3 Representative CE electropherograms indicating sialic acid and fucose-containing *O*-glycans as inferred after their loss upon acetic acid (AcOH) or fucosidase-treatments, respectively. Selected glycans are numbered as described in Fig. 3A.
