# Supplementary material for: Host responses to Clostridium perfringens challenge in a chicken model of chronic stress
Source: Gut Pathog. 2020 May 6;12:24. doi: 10.1186/s13099-020-00362-9 (PMC7203818; doi:10.1186/s13099-020-00362-9)
Supplement: Supplementary file 2 — Additional file 2. Additional tables. [file 13099_2020_362_MOESM2_ESM.pdf]

Host responses to *Clostridium perfringens* challenge in a chicken model of chronic stress

Sarah J.M. Zaytsoff,[a,b] Sarah M. Lyons,[c] Alexander M. Garner,[d] Richard R.E. Uwiera,[b] Wesley F. Zandberg,[c,e] D. Wade Abbott,[a] G. Douglas Inglis[a]#

[a]Agriculture and Agri-Food Canada, Lethbridge, Alberta, Canada

[b]Department of Agricultural, Food, and Nutritional Science, University of Alberta, Edmonton, Alberta, Canada

[c]Department of Biology, University of British Columbia (Okanagan Campus), Kelowna, British Columbia, Canada

[d]Department of Biochemistry, University of British Columbia (Okanagan Campus), Kelowna, British Columbia, Canada

[e]Department of Chemistry, University of British Columbia (Okanagan Campus), Kelowna, British Columbia, Canada

SUPPLEMENTAL MATERIAL

Table S1. Feed ingredients for 21% poultry starter diet

Ingredient List	Minerals	Vitamins
Wheat	Copper Sulfate	Vitamin A
Soybean meal	Ethylenediamine dihydroiodide	Vitamin D
Canola meal	Manganese Sulfate	Vitamin E
Corn distillers	Sodium Selenite	Vitamin K
Faba beans	Zinc Oxide	Thiamine monohydrate
L-Lysine HCl	Copper Proteinate	Pryidoxine
Enzyme	Manganese Proteinate	Calcium pantothenate
Choline chloride	Selenium Yeast	Niacin
ALIMET	Zinc Proteinate	Riboflavin
Limestone		Biotin
		Folic acid
		Vitamin B12
		Ethoxyquin

Table S2. List of primers used for qPCR and mRNA gene expression

Name	Abbreviation	Sequence 5' to 3'		Product Size	Reference Sequence	Source
C. perfringens (16S)	*CP1.2*	Forward	AAAGATGGCATCATCATTCAAC	283		PMID: 12399288
		Reverse	TACCGTCATTATCTTCCCCAAA			
NetB Toxin	*NetB*	Forward	AAATATACTTCTAGTGATACCGCTTCACA	78		PMID: 20457789
		Reverse	GAGGATCTTCAATAAATGTTCCACTTAA			
Toll-like receptor 2A	*TLR2A*	Forward	CAGCACAAGAGGCGTTCA	100	NM_204278.1	This Study
		Reverse	AACATTTTGGTGTAGCTGAGATG			
Toll-like receptor 15	*TLR15*	Forward	ACTAGAACCTGCCTGCCAAC	102	NM_001037835.1	This Study
		Reverse	AGCATCAGGAGAGATTGCCC			
Claudin 3	*CLDN3*	Forward	GGGATTTCTACAACCCGCTG	200	NM_204202.1	This Study
		Reverse	CTTGTCGTAGCTGGTAACGG			
Claudin 5	*CLDN5*	Forward	GGGGACTAAATGCAGAGACC	128	NM_204201.1	This Study
		Reverse	ATAGCCTAAGCATCACGAGC			
Occludin	*OCLN*	Forward	TGAATGCACCCACTGAGTGTT	99	NM_205128.1	This Study
		Reverse	CCAGAGGTGTGGGCCTTAC			
Mucin 2B	*MUC2B*	Forward	ATTGTGGTAACACCAACATTCATC	135		PMID: 23349743
		Reverse	CTTTATAATGTCAGCACCAACTTCTC			
Mucin 5AC	*MUC5AC*	Forward	TCCACCAGCTTCCAAATCCC	79	XM_015286693.1	This Study
		Reverse	GGGGTTGCCAGCCTTTACTT			
Interleukin 1β	*IL1*β	Forward	TGCCTGCAGAAGAAGCCTCG	137	NM_204524.1	This Study
		Reverse	CTCCGCAGCAGTTTGGTCAT			
Interleukin 2	*IL2*	Forward	TAACTGGGACACTGCCATGA	93	NM_204153.1	This Study
		Reverse	GATAGAGATGCTCCATAAGCTGT			
Interleukin 18	*IL18*	Forward	GTGAAGAGATCGCTGTGTGT	80	NM_204608.1	This Study
		Reverse	ATCGCATTCCAGCTCATCATC			
Interferon γ	*IFN*γ	Forward	GCTCCCGATGAACGACTTGA	121	NM_205149.1	This Study
		Reverse	GCATCTCCTCTGAGACTGGC			
Interleukin 6	*IL6*	Forward	AGGACGAGATGTGCAAGAAG	176	NM_204628.1	This Study
		Reverse	CATTTCTCCTCGTCGAAGCC			
Transforming Growth Factor β2	*TGF*β	Forward	CCATCTACAACAGCACCAGGG	157	NM_001031045.3	This Study
		Reverse	TAGCTTGGTGGGATGGCATTTTC			
Colony Stimulating Factor 3	*G-CSF*	Forward	AACTTATTTGTGCCCCCACG	84	NM_205279.1	This Study
		Reverse	ATACAGTACAAAACGCCGCC			
β-Actin	*BA*	Forward	CTCTGACTGACCGCGTTACT	172	NM_205518.1	This Study
		Reverse	TACCAACCATCACACCCTGAT			
Tata-Box Binding Protein	*TBP*	Forward	GTTCCCTGTGTCGCTTGC	147		PMID: 27685470
		Reverse	TAGCCCGATGATGCCGTAT			
